# Task shifting to improve the provision of integrated chronic care: realist evaluation of a lay health worker intervention in rural South Africa

**DOI:** 10.1136/bmjgh-2018-001084

**Published:** 2019-01-29

**Authors:** Felix Limbani, Margaret Thorogood, Francesc Xavier Gómez-Olivé, Chodziwadziwa Kabudula, Jane Goudge

**Affiliations:** 1 Centre for Health Policy, Faculty of Health Sciences, School of Public Health, University of the Witwatersrand, Johannesburg, South Africa; 2 Division of Health Sciences, University of Warwick, Coventry, UK; 3 MRC/Wits Rural Public Health and Health Transitions Research Unit (Agincourt), Faculty of Health Sciences, School of Public Health, University of the Witwatersrand, Johannesburg, South Africa

**Keywords:** task shifting, lay health worker, South Africa, realist evaluation, chronic care

## Abstract

**Introduction:**

Task shifting is a potential solution to the shortage of healthcare personnel in low/middle-income countries, but contextual factors often dilute its effectiveness. We report on a task shifting intervention using lay health workers to support clinic staff in providing chronic disease care in rural South Africa, where the HIV epidemic and an ageing population have increased demand for care.

**Methods:**

We conducted a realist evaluation in a cluster randomised controlled trial. We conducted observations in clinics, focus group discussions, in-depth interviews and patient exit interviews, and wrote weekly diaries to collect data.

**Results:**

All clinic managers had to cope with an increasing but variable patient load and unplanned staff shortages, insufficient space, poorly functioning equipment and erratic supply of drugs. These conditions inevitably generated tension among staff. Lay health workers relieved the staff of some of their tasks and improved care for patients, but in some cases the presence of the lay health worker generated conflict with other staff. Where managers were able to respond to the changing circumstances, and to contain tension among staff, facilities were better able to meet patient needs. This required facility managers to be flexible, consultative and willing to act on suggestions, sometimes from junior staff and patients. While all facilities experienced an erratic supply of drugs and poorly maintained equipment, facilities where there was effective management, teamwork and sufficient space had better chronic care processes and a higher proportion of patients attending on their appointed day.

**Conclusion:**

Lay health workers can be valuable members of a clinic team, and an important resource for managing increasing patient demand in primary healthcare. Task shifting will only be effective if clinic managers respond to the constantly changing system and contain conflict between staff. Strengthening facility-level management and leadership skills is a priority.

**Trial registration number:**

ISRCTN12128227.

**Key questions**

**What is already known?**

- Shortage of nurses in South Africa has been reported by the South African Nursing Council and in several other studies.
- Although task shifting is effective in addressing health worker shortage, there is limited evidence for task shifting from nurses to lay health workers (LHW) in integrated chronic care.

**What are the new findings?**

- We found that task shifting from nurses to LHWs improved the appointment systems, filing, prepacking medication, managing the chronic care pathway and patients’ adherence to their appointments, and so strengthened the functioning of primary healthcare clinics.
- While all facilities experienced an erratic supply of drugs and poorly maintained equipment, facilities where there was effective management, teamwork and sufficient space had a higher proportion of patients attending on their appointed day.

**What do the new findings imply?**

- The LHW intervention is a feasible, acceptable, sustainable and low-cost model that improves process outcomes, when implemented in clinics that are appropriately resourced (sufficient space, functional equipment, adequate supply of drugs and materials) and effective clinic management.

## Introduction

In low/middle-income countries (LMIC), task shifting, where tasks are transferred from more to less qualified cadres, is seen as a solution to the shortage of healthcare personnel.^1^ However, there are often factors that can dilute its effectiveness.^1^ The growing number of patients with a chronic condition in LMICs has led to an increase in patient load in primary healthcare clinics. While shifting some of the nurses’ tasks to lay health workers may relieve some of the pressure, there is little evidence on the effect of task shifting on the provision of chronic care. Existing evidence is focused on shifting tasks from doctors to nurses.^2^ Those studies which have considered the shifting of tasks to lay health workers have mainly concerned those working in communities rather than health facilities, and have focused on specific conditions such as HIV and AIDS, or maternal and child health.^3–5^

In nurse-led primary healthcare facilities in rural South Africa there has been an increase in demand for care due to the large number of people receiving HIV and tuberculosis (TB) treatment and the growing prevalence of non-communicable diseases in an ageing population. In 2011, the integrated chronic disease management initiative was intended to improve patient flow and speed patients’ journey through the clinic. However, the new process generated extra tasks for nurses (table 1), and there was evidence that nurses were struggling to manage the extra work, and, as a result, the initiative has often not been implemented as intended.^6^

**Table 1:** Nurses’ additional tasks under integrated chronic disease management

Additional task	Description
Appointment scheduling	Nurse sets date for the patient’s next appointment.
Preappointment retrieval of patients’ records	Patient files to be retrieved prior to a patient’s appointment. This is often done by nurses due to shortages in clerical staff.
Predispensing of chronic medication	Prior preparation of patient’s medication before the appointment.
Managing a separate queue for patients with chronic disease	A separate vital signs station* and a designated consultation room for patients with chronic disease, both staffed by nurses.

*The vital signs station, mostly located at the reception in view of other patients, is where a nurse measures blood pressure, temperature, pulse and weight for each patient attending the clinic.

In this paper, we report on a task shifting intervention in which lay health workers assisted nurses. We examined the extent to which task shifting happened and what factors affected the intervention’s effectiveness. We explored what contextual influences, and through what mechanisms, the lay health worker intervention enhanced integrated chronic care for patients with hypertension and modified patient outcomes in rural clinics in South Africa. This study was a realist evaluation carried out within the Nkateko cluster randomised controlled trial.^7^

### Our initial programme theory

**Figure 1:**
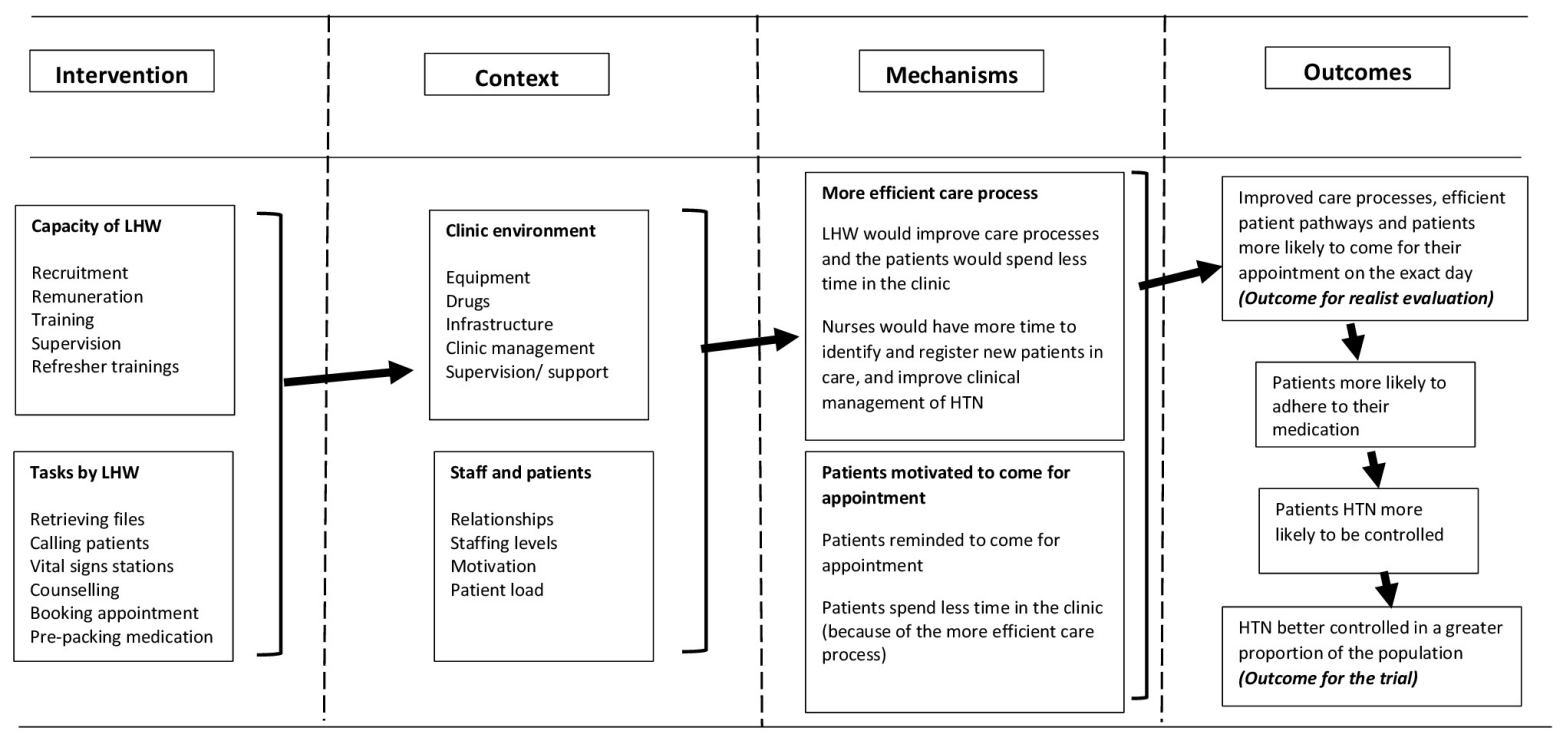
Initial programme theory of the lay health worker (LHW) intervention. HTN, hypertension.

We assumed that the high prevalence of uncontrolled blood pressure (BP) in the study communities is as a result of poor quality of care in the primary care clinics, which are overwhelmed by increasing number of patients with chronic conditions. By introducing lay health workers in the clinics, who are well trained, supervised and remunerated (figure 1; the intervention column), we hypothesised that this would free nurses to focus on the clinical management of patients, and to identify and register more patients into care (figure 1; the mechanisms column). Lay health workers would retrieve files, manage vital signs stations, book appointments, provide counselling, assist in prepacking medication and send messages or call patients to remind them of their appointment (figure 1; the intervention column). We anticipated that the more efficient care process and the reminders would motivate more patients to come for their appointment and increase the patients adhering to their medication. The overall result would lead to increased diagnosis and control of hypertension at a population level (see figure 1; outcomes column).

### The realist evaluation objectives

Our objective for the realist evaluation was to understand under what context and through what mechanisms a clinic-based lay health worker intervention would enhance integrated chronic care for patients with hypertension and would modify patient outcomes. We explored: how the intervention was developed and implemented in each clinic (process and fidelity); how functioning of the clinic and the provision of chronic care varied between the clinics before and during the study period (context); and what underlying mechanisms (causal processes) explained the effect of the intervention on clinic attendance (and other patient care outcomes) in different contexts.

Although we largely used the context-mechanisms-outcomes (CMO) configuration realist methodology to frame and analyse the paper, our understanding of the provision of chronic care was influenced by the Wagner model of effective chronic care^8^ and the theory of complex adaptive system.^9^ Wagner’s theory states that chronic care depends on a positive interaction between the health system, the providers and the users. The theory of complex adaptive system recognises that healthcare facilities are complex organisations and are always evolving and changing. A complex mix of relations, management and resources results in non-linear path of implementation and outcomes. This understanding helped us in shaping our programme theory.

### The provision of care for chronic conditions in primary healthcare clinics in South Africa

Primary care in South Africa is provided through 8-hour clinics and 24-hour community health centres. Staff comprise clinic operation managers, professional nurses, enrolled nurses, lay counsellors and data capturers. On attending a clinic, patients with chronic diseases have a dedicated queue. Each patient is given a personal file and have their vital signs measured (temperature, pulse, weight and BP). They go for consultation in a designated chronic care room for diagnosis, prescription and dispensation of medication. Although this is the intended process for every clinic, functioning of designated queues and consultation rooms depends on sufficient staff, equipment and space in the clinic.^10^

## Methods

### The randomised trial

The Nkateko pragmatic cluster randomised trial was conducted in seven rural primary care clinics and one rural health centre, and assessed the effect of placing two lay health workers in a clinic to support chronic disease care.^7^ Four facilities were randomised to receive this support; the remaining clinics operated as usual. After considering the structure and setup of primary healthcare, we designed the intervention so that it could realistically be rolled out in South Africa. Considerations included their levels of remuneration, who would be appropriate to supervise them and what tasks they could perform. The lay health workers reported to the clinic manager and were paid the same rate as other community health workers. (Lay health workers have similar education levels to those who work in the community.^1^) The lay health workers were supported and supervised by the implementation manager, an experienced local nurse, employed by the project. She recruited and trained the lay health workers, provided on-the-job training and supportive supervision. During the development phase of the trial, she held workshops with each individual intervention clinic to determine what tasks the lay health workers would undertake. If the intervention were to be rolled out, we envisaged that the implementation manager’s role could be performed by the clinic supervisors, who are responsible for four to five clinics. At the end of the intervention there was no difference in the primary outcome of the trial, which was prevalence of clinic users at moderate or higher risk of cardiovascular disease as assessed by two population surveys.^10^

### The realist evaluation

Given the growing focus on the role of context and causal mechanisms in order to understand complex health service programmes, we decided to undertake a realist evaluation to supplement trial methodology by providing additional information that would explain the trial outcomes. The evaluation was conducted in the eight trial clinics for 4 months prior to the start of trial, and then continued throughout the intervention. We took a case study approach, with each clinic being one case, to compare how tasks were conducted with and without the lay health workers. We asked under what circumstances the task shifting did work, for whom and why.^11^ The realist CMO framework focused our attention on how context influenced participants’ engagement with the intervention (the mechanism). The outcome for the realist evaluation was the proportion of patients who attended on their appointed day, a more proximal outcome than population-level management of hypertension (figure 1). We also explored how the intervention influenced patient pathways and activities associated with the provision of chronic care. However, we were not able to examine the extent of BP control among clinic attendees; although we extracted these data from the patients, the clinic BP machines were not maintained and therefore not sufficiently accurate (see below) for these data to be used as an outcome. Nor were we able to examine the number of new patients enrolled in care in the control clinics as this would have meant disrupting the work flow of the clinic. We did gather this information in the intervention clinics; it will be reported in a forthcoming paper.

### Study site and population

The study was conducted in a health and demographic surveillance system (HDSS) site run by the Medical Research Council/Wits Rural Public Health and Health Transitions Research Unit based in Bushbuckridge, a poorly resourced rural subdistrict of Mpumalanga province, north-east South Africa.^12^ At the time of this study, the HDSS site covered 31 villages, 20 000 households and 115 000 individuals. Ten primary healthcare facilities and three hospitals (that are 25–60 km away from the site) served the local population in the study site.

### Data collection

We observed clinic activities and the patient pathway (The route a patient with chronic disease will take from first to the last contact with a clinic staff. It included receiving files, having vital signs measured (including BP), consultation and booking for the next appointment), and conducted interviews (see table 2 and data collection tools presented in online supplementary material). Sampling was purposeful to ensure representation of a range of views. On the days that clinic observations were held, 10 patients, chosen at different points during the day, were interviewed as they left the clinic. From May 2014 to July 2015, a clerk placed in each of the eight clinics, collected identification data and clinical details of attendees, allowing us to assess clinic workloads. The implementation manager and the author FL kept weekly field diaries.

10.1136/bmjgh-2018-001084.supp1 Supplementary data

**Table 2:** Qualitative data collection, participants and frequency of contacts

Method	Participants	Frequency of contacts	Data collected
Observations	Clinic activities	240 observation days	Descriptions of clinic activities, operation of intervention activities, patient pathways and barriers and facilitators to chronic care and how they changed over time.
	Patient consultations	443 patient consultations	Descriptions of nurses, lay health workers and patients’ interactions, and how they changed over time. We assessed time spent by patients during consultations to understand nurse workload and how the time spent affects adherence of patients to appointment.
In-depth interviews	Clinic managers	36 interviews with 9 managers (4 per participant)	Exploring clinic routines, their expectations of the research, any concerns, how patients with chronic diseases are managed, their experience with the lay health worker intervention and perceptions of whether change occurred.
	Clinic supervisors	6 interviews with 3 supervisors (2 per supervisor)	
	Subdistrict manager	3 interviews	
	Lay health workers	60 interviews with 10 workers (6 per worker)	Monthly account of implementation progress. Details of day-to-day experience in the clinic and how the clinics were changing.
	Implementation manager	9 interviews	Progress of the intervention across the clinics and how differently the intervention was running from one clinic to another.
Exit interviews	Patients	703 interviews	Patients’ experience of care in the clinic and their engagement with the LHWs, nurses and staff in general, and how this changed with the intervention.

LHW, lay health worker.

We had four periods of data collection: preintervention; the introductory phase of the intervention; mid-way through the implementation; and at the end of the intervention. This was helpful in understanding how the intervention unfolded and factors that affected its implementation process.

The first author’s background in health policy research and better understanding of health dynamics in Southern Africa rural context helped to better engage the interviewers, clinic staff and patients. Three fieldworkers with qualitative and quantitative experience received an initial week-long training in research methods, ethics and the study tools that we used. The fieldworkers were fluent in Shangaan (the local language) and English. The fieldworkers conducted clinic observations, observation of patient consultation and patient exit structured interviews. A fourth fieldworker was responsible for translation and transcription. Eight data entry clerks (one per clinic) were responsible for collecting patient record data and reported to the Agincourt HDSS data manager. The first author conducted in-depth interviews with lay health workers, the implementation manager and health personnel.

Qualitative data from interviews with lay health workers, implementation manager, clinic managers, supervisors and the subdistrict manager were tape recorded, translated and transcribed into English verbatim. Data from clinic observations were captured as field notes in line with an observation guide. Field note books, tapes with data and electronic databases are stored at Agincourt data archive.

### Data analysis

We drew together the different sources of data for each facility, distilling data into data extraction tables in Microsoft Word, using inductive and deductive approaches to identify the informative text, either summarising descriptions of events, or extracting raw data such as useful quotations.^13^ In the data extraction table, each data source had its own row and page numbers were recorded so as to be able to go back to the original data. And the data sources (and so rows) were ordered chronologically to enable us to see change over time. Quantitative data from patient exit interviews and clinic use were analysed descriptively using simple statistical tests.

The extracted data for each clinic allowed us to develop within and across-clinic comparative case analyses to explain and interpret outcomes. Author FL did the data extraction and reviewed the tables alongside the quantitative data for each individual clinic, formulating the preliminary explanatory theories at the individual clinic level. He then compared the theories across different clinics to identify similarities and differences, returning to the data extraction tables and the original data to clarify findings. Author JG and MT reviewed the theories and the data, comparing different clinics to refine theories of how the intervention worked (or not) through particular mechanisms in particular contexts (expressed as CMO configurations). Revisiting the data throughout the process enabled us to confirm descriptions of the context, enrich the content, provide clarity and check emergent ideas.

## Results

### The intervention

In the intervention clinics, the lay health workers measured BPs, booked appointments, retrieved files a day before the appointment and refiled afterwards, provided health education and assisted nurses with prepacking of medication. They also reminded patients of their appointments and followed up with those patients who missed an appointment. We planned that the reminders would be text (short message service) messages, but the lay health workers found this was not effective as older people could not see or read the text, phones were often shared and there was no indication of whether a text message had been received. They therefore changed to using direct phone calls.

In addition, lay health workers became involved in a series of other tasks. These tasks included measuring weight, height, temperature and other vital signs; weighing babies and pregnant women; measuring blood glucose; testing urine; collecting sputum bottles from consultation rooms and storing them in the refrigerator; and helping in the dressing of wounds. They also opened files for new patients, typed clinic documents, registered pregnant women on ‘mom connect’ (an mHealth initiative by the Department of Health) and helped nurses to compile monthly data reports. Despite these additional tasks, the lay health workers managed to complete the original set of tasks.

### The outcome

**Figure 2:**
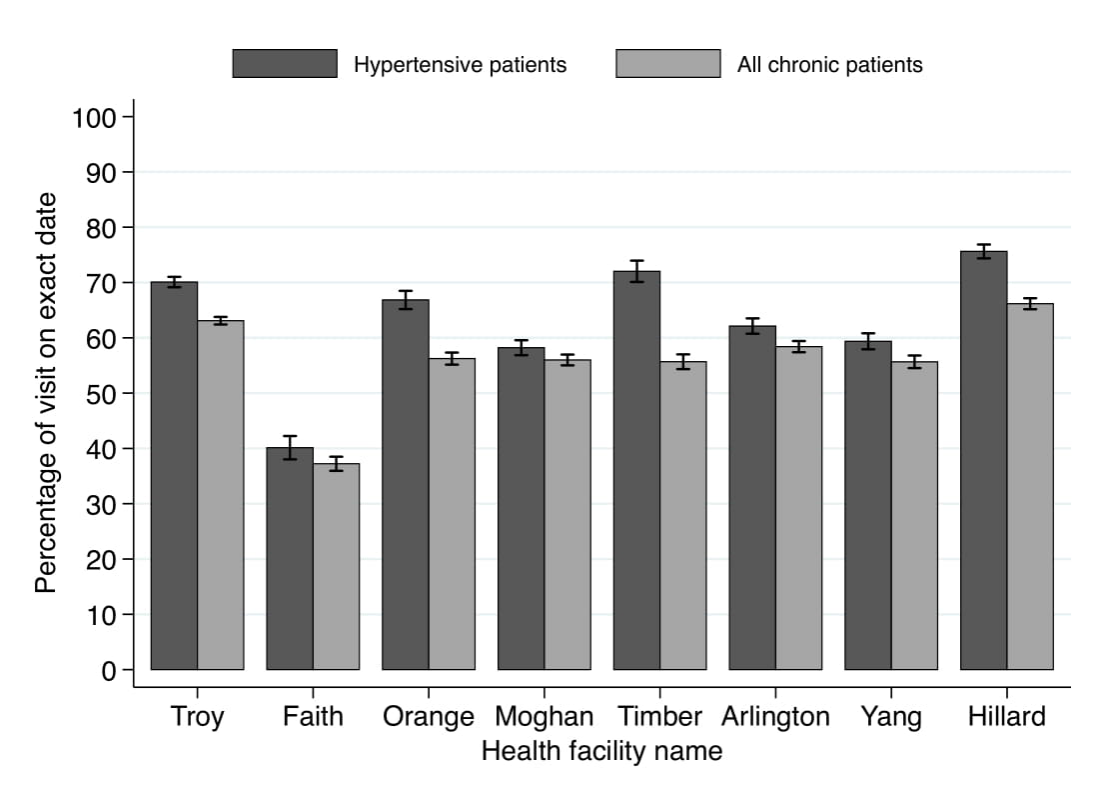
Adherence to appointment dates (May 2014 to July 2015).

In the intervention clinics over 70% of patients with hypertension attended on the correct day, compared with 54% of patients with hypertension in the control clinics (figure 2), indicating a positive effect of the intervention.

People with hypertension were more likely to attend appointments on exact dates (because of the reminders by lay health workers) than the rest of patients with chronic diseases (table 3). This was particularly true of older rather than younger people, and younger females than younger males (P≤0.05). Most of the non-hypertensive patients attended for HIV treatment.

**Table 3:** Patient attendance on appointment dates (May 2014 to July 2015)

Age group	Hypertensive				Non-hypertensive			
	Male		Female		Male		Female	
	n	%	n	%	n	%	n	%
18–29	60	49	173	58	530	51	2716	49
30–49	800	58	3535	63	2648	48	7772	54
50+	3832	67	14 033	65	1382	53	2265	55
Total	4692	65	17 741	65	4560	49	12 753	53

In the intervention clinics the tasks of booking appointments, prepacking medication and retrieving patients’ files were completed more often, in comparison to the control clinics (tables 4 and 5).

Managing chronic patients before the lay health workers was so difficult. Nurses are few yet they did filing, measured vital signs, gave health education, counselling, identified cases of elevated blood pressure or sugar, and checked appointment cards of patients. The number of chronic patients in a day was always high. Now, most of these tasks are done by the lay health workers. Patients are no longer missing appointments, their files are pre-retrieved and their medication prepacked. They spend less time in the clinic. (Manager, Hillard clinic)

The waiting time for patients in the clinics with lay health workers fell by around 1 hour during the intervention, from around 3 hours 20 min to around 2 hours 25 min. The poorest performing intervention clinic (Orange) had higher clinic attendance than the best performing control clinic (Arlington) because of the appointment reminders.

**Table 4:** Clinic context, mechanisms and outcome configuration for intervention clinics

Clinic name	Context							Mechanisms		Outcomes		
	Clinic infrastructure	BP machines	Other materials (files, drugs, packs for drugs)	Clinic management	Visit per nurse per month (% change)	Existing manager, staff and patient relationship	LHW intervention	Chronic care processes	Patients adhering to appointments	Chronic care pathway	Other aspects of chronic care	Patient attending on booked date
Hillard	Modern spacious building	Broke down a few times	Erratic supply	Strong clinic manager in control of the clinic	528 to 429 (−19%)	Good relationship among staff and with patients	Skilled LHWs. 36% workload increase per month. Good teamwork, supported by staff.	Strong manager led to good relations for staff. Better equipment. Low patient load motivated staff to be dedicated.	Space facilitated quality counselling and confidential space. Skilled LHWs, dedicated queue led to lesser clinic time.	*Functional*: chronic care pathway *Erratic*: chronic consultation room *Did not happen*: designated vital signs station	*Functional*: filing, appointments, prepacking medication, phone call reminders	HTN: 76% (n=4482) Other chronic: 66%
Timber	Modern building but with limited space	Frequently broke down	Erratic supply	Strong clinic manager but not liked by nurses	433 to 202 (−53%)	Poor relations among staff and with patients	Skilled LHW. 7% workload decrease per month. Good teamwork, supported by staff.	Nurses frustrated with breakdown of equipment and limited space. This led to lack of commitment and poor relations.	Skilled LHWs ensured patients attend appointment, yet affected by lack of space and nurses were frustrated.	*Functional*: chronic consultation room *Erratic*: designated vital signs station *Did not happen*: chronic care pathway	*Functional*: filing, appointments, phone call reminders *Erratic*: Prepacking medication	HTN: 72% (n=2035) Other chronic: 56%
Troy	Modern building but with limited space	Frequently broke down	Good supply of materials	Weak clinic manager	252–347 (+38%)	Poor relations among staff and with patients	Skilled LHW. 48% workload increase per month. Good teamwork, supported by very few staff.	Weak manager, limited space and BP machines that broke down led to poor relations and staff not willing to work.	Skilled LHW that ensured patients attend appointment, yet patients did not like staff attitude and long waiting.	*Erratic*: chronic consultation room, chronic care pathway *Did not happen*: designated vital signs station	*Functional*: filing, appointments, phone call reminders *Erratic*: prepacking medication	HTN: 70% (n=8960) Other chronic: 63%
Orange	Dilapidated building with limited space	Frequently broke down	Erratic supply	Weak clinic manager	276–413 (+50%)	Poor relations among staff and with patients	Unskilled LHW. 79% workload increase per month. Poor teamwork, supported by very few staff.	Weak manager, poor infrastructure, limited space and BP machines that broke down led to poor relations and staff not willing to work.	Poor queuing, infrastructure, unskilled and uncoordinated LHWs led to long queues and patients unsatisfied with care services.	*Functional*: chronic consultation room *Erratic*: chronic care pathway *Did not happen*: designated vital signs station	*Functional*: phone call reminders, filing, appointments but with some mistakes. *Did not happen*: prepacking of medication	HTN: 67% (n=3123) Other chronic: 56%

HTN: patients with hypertension; other chronic: patients without hypertension.

BP, blood pressure; LHW, lay health worker.

**Table 5:** Clinic context, mechanisms and outcome configuration for control clinics

Clinic name	Context						Mechanisms		Outcomes		
	Clinic infrastructure	BP machines	Other materials (files, drugs, packs for drugs)	Clinic management	Visit per nurse per month (% change)	Existing manager, staff and patient relationship	Chronic care processes	Patients adhering to appointments	Chronic care pathway	Other aspects of chronic care	Patient attendance on booked date
Arlington	Modern spacious building	Did not break down	Erratic supply	Strong manager but often absent from clinic	359–370 (+3%)	Very poor relations among staff and with patients	Better equipment and strong manager but failed to motivate staff because of absenteeism. This led to poor staff relations, lack of support to each other and taking long to attend to patients.	Spacious building facilitated a separate consultation room but poor staff relations led to poor commitment. Patients always complained of poor staff conduct.	*Functional*: chronic care pathway, chronic consultation room *Did* *n* *o* *t happen*: designated vital signs station	*Functional*: filing *Erratic*: appointments *Did* *n* *o* *t happen*: prepacking of medication	HTN: 62% (n=4618) Other chronic: 58%
Yang	Dilapidated building, limited space	Did not break down	Erratic supply	Strong manager replaced by weak manager	306–342 (+12%)	Good relationship among staff and with patients	Initial strong manager and better equipment motivated staff relations, committed staff and quick services to the satisfaction of patients. Later, weak manager and lack of support to staff.	Dilapidated building led to poor queuing and lack of confidential consultation. Patients’ files often missed and patients were frustrated with the little support they received.	*Erratic*: chronic consultation room *Did* *n* *o* *t happen*: chronic care pathway, designated vital signs station	*Erratic*: appointments, filing and prepacking medication	HTN: 59% (n=4347) Other chronic: 56%
Morgan	Dilapidated building, limited space	Did not break down	Erratic supply	Weak clinic manager	374–447 (+20%)	Good relationship among staff and with patients	Weak manager that failed to discipline and control staff. This led to staff behaving as they wished sometimes, that is, arriving late or leaving work early.	Good relation among staff resulted in effective support to patients. Limited space led to poor queuing with patients often quarrelling.	*Functional*: chronic consultation room *Erratic*: chronic care pathway *Did* *n* *o* *t happen*: designated vital signs station	*Functional*: appointments *Erratic*: filing *Did* *n* *o* *t happen*: prepacking of medication	HTN: 58% (n=5039) Other chronic: 56%
Faith	Modern spacious building	Frequently broke down	Erratic supply	Weak manager	372 to 227 (−40%)	Poor relations among staff and with patients	Weak management that resulted in poor relations among staff and between staff and patients. Staff felt unsupported and were not willing to work. Patients were often ignored.	Spacious infrastructure facilitated confidential consultations and designated queue sometimes. Poor filing system led to long waiting time and missing appointments.	*Functional*: chronic consultation room, chronic care pathway *Did* *n* *o* *t happen*: designated vital signs station	*Erratic*: filing, appointments *D* *id* *n* *o* *t happen*: prepacking of medication	HTN: 40% (n=2061) Other chronic: 37%

HTN: patients with hypertension; other chronic: patients without hypertension.

BP, blood pressure.

### The context

Prior to the start of the trial, seven of the clinics did not have designated vital signs stations for patients with chronic disease due to shortages of staff. There was no functioning appointment system in three clinics, and in four clinics, patient files were not retrieved in advance. Most of the clinics did not prepack medication because the medication had to be unpacked when patients did not arrive within a few days of their appointment.

Nurses are supposed to book and counsel patients. She starts filling the appointment register, then patients would come requiring her attention. She would leave that and attend to patients. Then that is not completed till the next day. The next day there are also patients to be booked and the work keeps on piling up. (Manager, Yang clinic)

Visits by patients with chronic diseases increased by almost 75% in both the intervention and control clinics during the intervention. This increase was due to the roll-out of antiretroviral therapy, an ageing population with a high prevalence of hypertension and local hospitals referring stable patients back to primary care. Appointments are for a specified day but no time is stated; patients often start queuing at 06:00 hours, although the clinic opens at 07:00 hours. As a result of the long queue, on a busy day nurses will skip their lunch break and work through to 14:00 hours until all patients have been seen. All the clinics had high turnover of staff, with variable effects on overall staffing levels (tables 4 and 5). Clinic managers complained that most of the new nurses were either newly qualified or had come from hospitals and were not yet familiar with the work of primary care clinics.

Due to the large patient load, and hence the heavy demand on the electronic BP machines, these machines often broke down and were rarely repaired: ‘*There is no system in place for servicing or maintenance. We once took some of the equipment to the hospital for maintenance, and until now, nothing has come back’* (Manager, Troy clinic). When the electronic BP machines broke down, nurses refused to use the mercury sphygmomanometers, because prolonged use of a stethoscope (up to 2 hours) hurt their ears. As the cuffs were also in poor repair (some had even been stapled or sewn together), we replaced them, but the new ones had worn out by the end of the 18-month intervention: ‘*S* *ometimes when they measure our BP they tell us that—all of you today your blood pressure is too high, maybe it’s the machine that is having a problem*’ (Patient with hypertension, Troy clinic). Patient files and bags for prepacking medication were also in short supply and the availability of hypertensive medication was erratic.

Five of the eight clinic buildings were in a dilapidated state, often with insufficient space for the growing number of patients, who, in some clinics, had to queue outside the clinic. ‘*Every day, we have four or five new stable (chronic disease) patients returning from X hospital. We were supposed to have two consultation rooms for chronic patients, but the clinic is small and doesn’t have sufficient rooms* *’* (Manager, Yang clinic). Space was so limited in one clinic that sometimes two consultations took place at the same time in the same room. In three of the four intervention clinics, the lay health workers were stationed in the waiting area, a corridor or a small room, used for other purposes. None of these locations were suitable for confidential conversations with patients.

Two of the intervention clinics had lay health workers who performed exceptionally well (table 4: Hillard and Timber). They were innovative, committed, hardworking and supported each other. They initiated calling patients when sending text messages proved ineffective. Their appointment records, filing and prepacking medication were kept up to date. There were similar levels of skills and teamwork among the lay health workers in a third clinic but the high patient load affected their performance, so the project hired a third worker. In the fourth clinic, the lay health workers performed less well. With little support from the clinic staff, they were slow, and worked in isolation, making mistakes in the appointment and filing systems. Of the intervention clinics, this clinic had the lowest proportion of patients with hypertension who came on their appointed day (table 4).

In part due to these problems and the long patient queues, tensions in the clinics often ran high. In some clinics, managers failed to contain this tension, leading to poor discipline among some staff who refused to take instructions from those in authority. ‘*The deputy clinic manager approached a nurse responsible for TB patients requesting her to consult acute patients. The nurse refused outright saying that other nurses shun TB patients’* (Observation notes, Troy clinic).

In other instances, nurses were leaving early, arriving late at work or taking long break periods. ‘*Three nurses were socially chatting in the consultation room and patients were left unattended to for about 45 min’* (Observation notes, Moghan clinic). ‘*Some nurses wondered how the clinic manager allowed other nurses to come for work without uniform and sometimes just leave for home without telling the clinic manager. Nurses felt the clinic manager was poor in managing staff*’ (Observation notes, Moghan clinic). These absences exacerbated the shortage of staff. In other facilities, managers maintained control by shouting, with the result that nurses were only committed to work when the manager was present.

Poorly managed staff engaged poorly with patients (eg, neglecting patients while they do personal things or criticising patients for coming in the afternoon although the clinic was open), which in turn affected the clinic operations. ‘*When a patient comes in the afternoon, nurses tell the patient to go back and come the following day. I feel bad for the patients*’ (Lay health worker, Orange clinic).

In clinics that were better run, managers were able to address the challenges, engendering a more positive attitude, and ensuring that both administrative work and patient care ran smoothly. Staff spoke politely to one another and the patients worked as a team and assisted one another. The intervention’s implementation manager made the following observation about one manager: ‘*She is a very good leader. She doesn’t have to go around following people to make things happen*’ (Implementation manager).

### Mechanism 1: more efficient care processes

Professional nurses generally appreciated the role of the lay health workers, recognising their skills and consulting with them as peers. One clinic manager noted that they were hardworking:

They are always doing their job. I can tell you there is a nurse here who always wants you to tell her what to do. But with the lay health workers, that is not the case. When they come on duty, they are the earliest. I wish they were nurses. They have good communication skills, especially the smile that they give to the patients. (Manager, Timber clinic)

However, some junior staff (enrolled nurses, enrolled nursing assistants and lay counsellors) were antagonistic towards the lay health workers perhaps because their professional standing was implicitly challenged when the lay health workers were asked to do similar tasks. For example, a lay counsellor resented being asked to take vital signs when the lay health worker was not there (Hillard clinic). In two clinics, junior nurses left vital signs stations to be managed by the lay health worker, until the clinic manager said that they should work together. A fieldworker observed an enrolled nurse rudely telling the lay health worker to familiarise herself with clinic procedures (Orange clinic). In Orange clinic, the lay health worker arrived early and started the morning’s health talk as soon as she could in order not to keep patients waiting. However, a junior nurse who arrived later accused her of monopolising the health talk, although the nurse showed no concern about keeping patients waiting. One nurse remarked: ‘*W* *e don’t need to hurry because the patients are keeping us company*’ (Orange clinic).

A well-managed clinic proved an ideal environment for task shifting. For example, in Hillard, the clinic manager was proactive in finding working space for the lay health workers. She included the lay health workers in staff meetings. She held meetings to explain the patient pathway to new staff, and, if necessary, explained to the waiting patients why appointments were taking a bit longer on a particular day. Nurses and lay health workers were proactive in designing ways to improve the service. For example, the lay health workers used quiet days to pull the patient files out several days in advance to ensure the process moved as fast as possible when the clinic was busy. As the nurses often had to leave their consultation room to collect medication from the pharmacy, the lay health worker suggested they give the medication to the patient as she/he entered the consultation room. This was tried for several days, until the patients expressed their concern about confidentiality. It was then agreed that the lay health worker would take medication for five patients at a time into the consultation room for the nurse.

In contrast in Troy, the problem of time spent handing out medication was solved by asking patients to collect their medication at the pharmacy window. While this no longer required the nurse to collect the medication, it also meant that the lay health workers were no longer able to provide counselling on how to take the drugs when patients received their medication. The service had been adapted to suit the nurses without taking into account the quality of the service. In Orange, staff abandoned the prepacking of medication, avoided doing morning health talks and avoided tasks requested by senior colleagues. The manager seemed to be passive. Lay health workers were not invited to the staff meetings, felt excluded and so were less able to resolve problems. ‘*I want to see our relationship with the nurses improving. They should invite us to the staff meetings. Sometimes nurses just place the files anywhere in the consultation room. During the next appointment, we struggle to find the files*’ (Lay health worker, Orange clinic).

Neither the best performing (Hillard) nor the least performing (Orange) intervention clinic maintained the separate vital signs station. Orange was able to maintain a separate consultation room, but not the pathway for patients with chronic diseases; Hillard maintained the patient pathway but not a separate consultation room. Both clinics experienced similarly erratic supply of resources and poorly maintained equipment. The difference between the two clinics was that Hillard was a modern spacious building, with responsive and effective manager, good relationships between staff, skilled lay health workers and a reduction in workload over the study period. In contrast, the Orange building was dilapidated, relationships between staff were poor, staff did not actively engage in improving how things were run and the lay health workers were less capable than others and had to cope with an increase in workload. It was the management skills, relationships between staff and the conducive environment of a more appropriate building that enabled the better performing clinic to take greater advantage of lay health workers.

**Figure 3:**
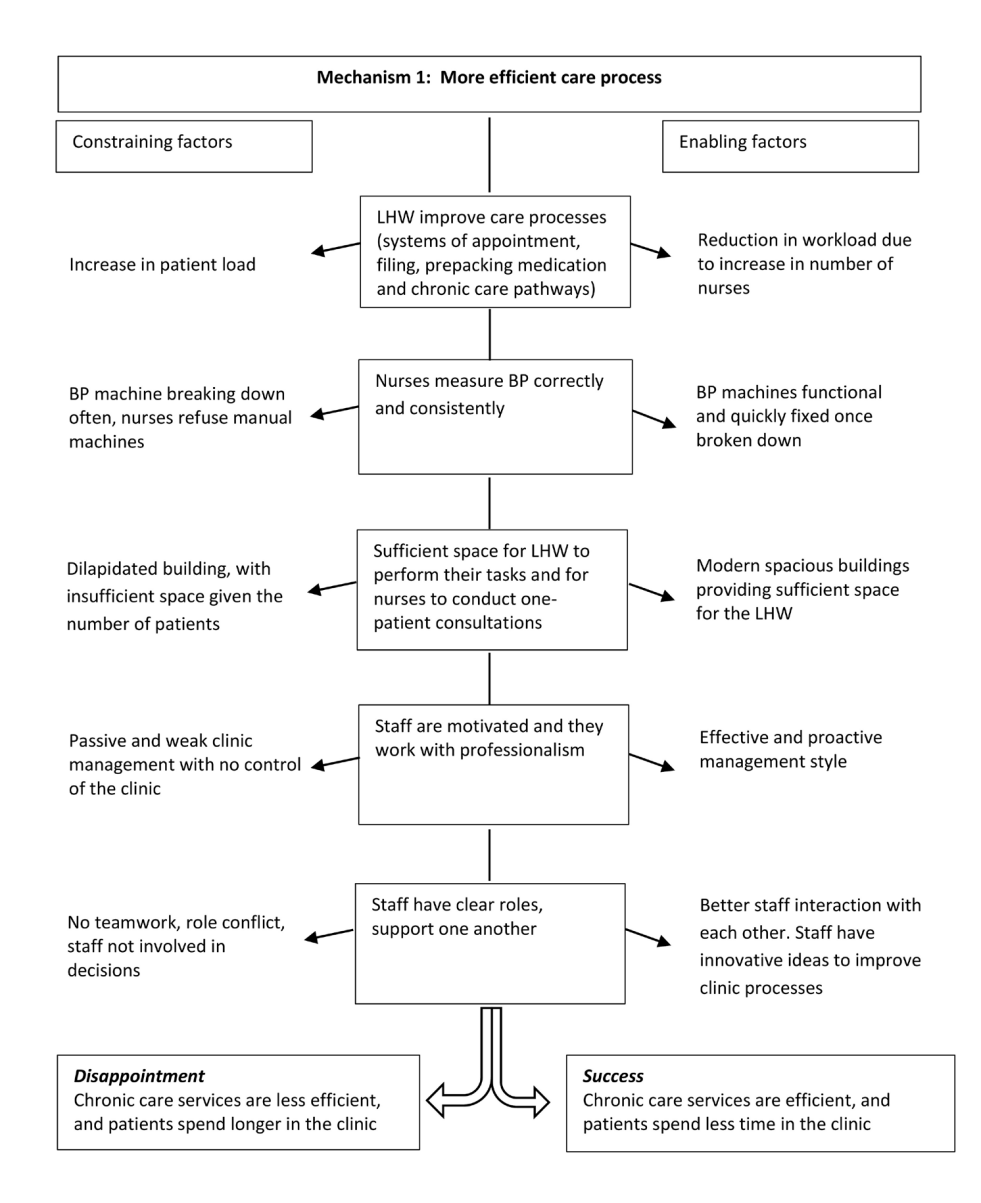
Mechanism 1—more efficient care processes. BP, blood pressure; LHW, lay health worker.

Figure 3 shows the more detailed programme theory in the central column of the diagram, derived from realist evaluation. The constraining factors (shown on the left) are the increase in patients, non-functioning BP machines, insufficient space, poor clinic management and little teamwork that leads to an inefficient care process and patients spending longer in the clinic. The enabling factors (shown on the right) are a reduction in workload due to an increase in nurses, functional BP machines, sufficient space, proactive management and better teamwork with the staff producing positive innovations in the care process.

### Mechanism 2: patients motivated to come for their appointments

The lay health workers were mostly welcomed by patients, who appreciated someone with a friendly face, who was willing to take time to talk to them. Both patients and nurses believed that the reminders encouraged patients to adhere to their appointment dates: ‘*I used to easily forget my appointment at the clinic. Nurses would become angry and shout at me for missing my appointment. But ever since the lay health workers started calling to remind me, I don’t miss my appointment*’ (Patient with hypertension, Timber clinic). On occasions, patients expressed irritation that they had been called to come to the clinic for their appointment when their medication was not available, due to drug outages. The patients felt that the lay health workers should have checked the drug stocks first. Some patients were concerned about confidentiality, as the lay health workers were from the local community. ‘*Most of the patients know us. When the ART patients find us here, they try to hide and yet they are supposed to pass by our station. It’s because of stigma and they don’t want to be noticed*’ (Lay health worker, Troy clinic).

Although dedicated queues for patients with chronic diseases facilitated faster queues for the patients, pathways for patients with chronic diseases were unstable and confusing across all the trial clinics. The pathways largely depended on the clinic infrastructure, adequate space in the clinic, availability of adequate and functional equipment and adequate number of nurses available in the clinic on a particular day. Troy was one of the clinics that managed a separate vital signs station for patients with chronic diseases: ‘*The coming in of lay health workers has helped us maintain a vital signs station for patients with chronic diseases. This has reduced the waiting time for the patients as patients are now attended to and get their medication faster than before’* (Manager, Troy clinic). However, in clinics like Moghan where space was very limited, staff and patients were concerned with challenges associated with queuing; ‘*Limited space is our biggest challenge. There is only one waiting area for both acute and chronic patients. We don’t have enough space for patients with chronic diseases to have their own vital signs station*’ (Manager, Moghan clinic*).* ‘*I don’t like it when there are a lot of patients and there is no space at the clinic. Sometimes we have to stand outside until there is space for us to sit inside the clinic’* (Patient with hypertension, Moghan clinic).

**Figure 4:**
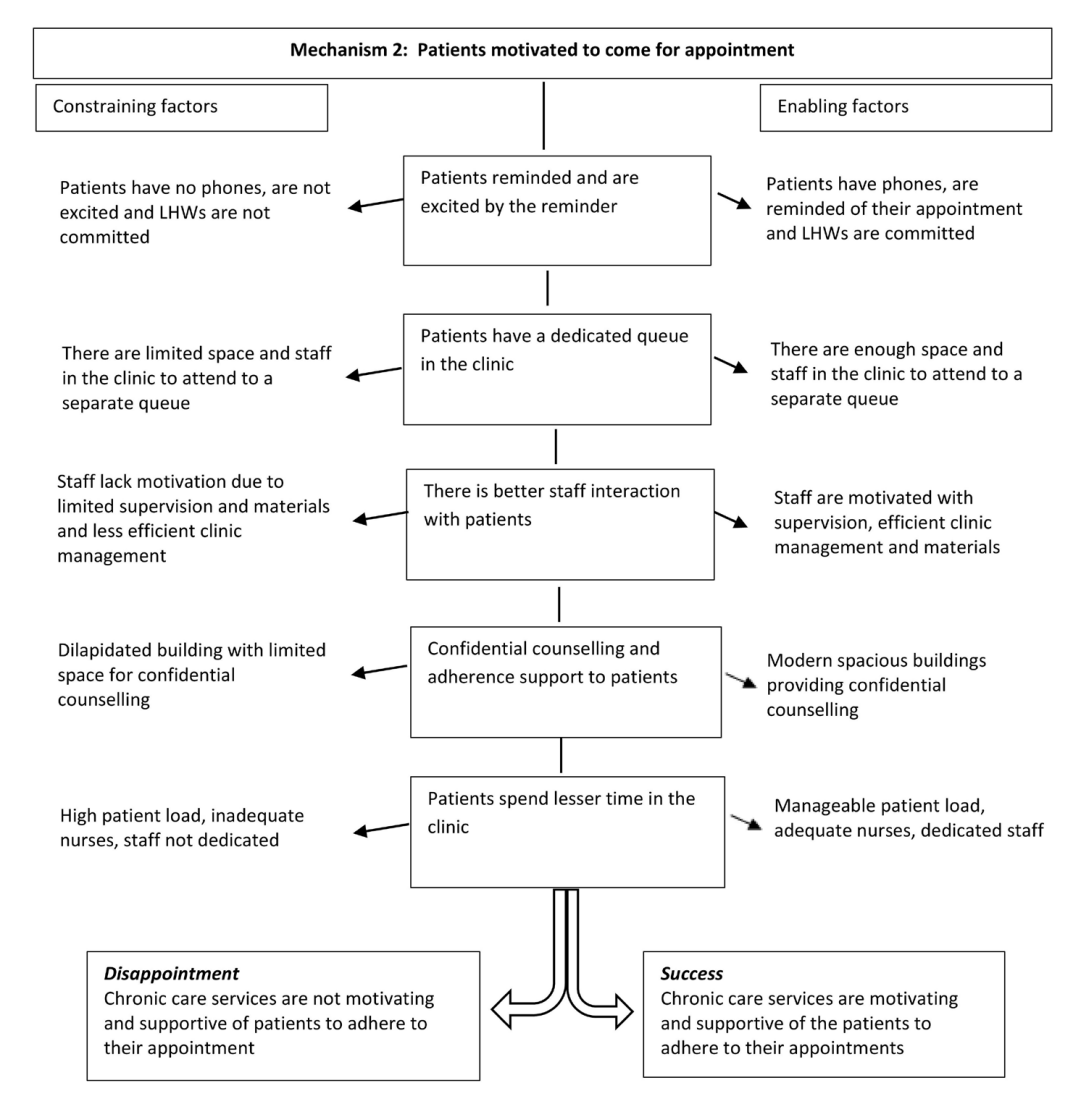
Mechanism 2—patients motivated to come for their appointments. LHW, lay health worker.

Figure 4 also shows the more detailed programme theory in the central column of the diagram, derived from realist evaluation, related to patients’ motivation to come for their appointment. The constraining factors (shown on the left) were instances where patients had no phones, were not excited with the reminders and lay health workers lacked commitment to remind the patients, limited space and staff in the clinic to manage a separate chronic queue and limited supervision and material support for staff that leads to chronic care services that are unsupportive of the patients to adhere to their appointment. The enabling factors (shown on the right) were instances when patients had phones, lay health workers were committed in reminding the patients, adequate space and staff to manage separate chronic queue and adequate supervision and material support for staff.

## Discussion

We have shown how the addition of lay health workers improved care for patients in intervention clinics compared with clinics without such support. All eight clinic managers had to cope with an increasing but variable patient load and unplanned staff shortages, often with insufficient space, poorly functioning equipment and an erratic supply of drugs. These conditions inevitably generated tension among staff. The introduction of the lay health workers in the four intervention clinics relieved the staff of some of their tasks and ensured previously neglected tasks were completed, but in some cases the presence of the lay health worker generated conflict with other staff.

Where managers were able to both respond to the changing circumstances, and to contain tension among staff, facilities were better able to meet patient needs. This required facility managers to be flexible, consultative and willing to act on suggestions, sometimes from junior staff and patients. Where managers were not responsive to the changing circumstances, they struggled to contain the tensions which then spilled over into interactions with patients. Staff shortages were exacerbated by poorer performance of some staff. While all facilities experienced an erratic supply of drugs and poorly maintained equipment, facilities where there was effective management, teamwork and sufficient space had a higher proportion of patients attending on their appointed day.

Studies have identified barriers to task shifting, such as weak management, poor relationships with other healthcare workers, resistance from higher level cadres who feel their skill is being threatened and inadequate supply of commodities.^14^ We observed all these problems. Moreover, many of the contextual problems have been noted in other South African studies,^15–17^ for example, the malfunctioning of the BP machines, a shortage of healthcare workers with overwhelming workload, the irregular supply of drugs and inadequate workspace,^15 18–20^ as well as issues such as insubordination, lack of professionalism and avoidable mistakes made by staff.

While lay health workers can be trained to do many tasks effectively, reduce the work load of nurses and so change the way the clinic runs, the addition of lay health workers to the clinic team will not solve the systemic problems (such as resources and supplies) that prevent effective care. However, they do have the potential to be valuable members of the team. To ensure their effectiveness is not constrained, managers must address issues of space and equipment, ensure their role is well understood, that junior staff are comfortable and not threatened and that ‘role creep’ does not overwhelm the lay health workers. The addition of lay health workers to clinic staff is a feasible and affordable intervention which can be realistically rolled out in South Africa; the rural clinics where this study was conducted are similar to other settings across South Africa.

In Box 1 we propose the key elements necessary for successful task shifting from nurses to lay health workers.

Box 1 **Priority list for successful task shifting from nurses to lay health workers in primary healthcare facilities**

**Host institution**

- Effective clinic manager who is positive about the value of lay health workers, and/or senior staff who are supportive to the lay health workers.
- Effective teamwork and good relationships among staff.
- Team members in the clinics should have clear job descriptions to avoid conflicts among staff.
- Careful management of the introduction of the lay health workers to reduce a potential role conflict.

**Health system**

- Reliable supply and maintenance of necessary equipment.
- Adequate and consistent supply of medication, and other essential materials (eg, files).
- Adequate clinical staff to carry out the clinical work, once relieved of other duties.
- Sufficient space in the clinic to ensure lay health workers have adequate space to work in.
- Clinic managers to be trained in managing staff and should undergo other leadership development programmes.

**Lay health workers**

- Skilled, innovative and hardworking lay health workers who work as a team.
- Supportive supervision by a health service manager in charge of the lay health workers who is able to provide on-the-job training.

Health systems are complex with often a non-linear path between implementation and outcomes^9^; our mixed methods approach and comparison of the different clinics allowed us to determine which factors were most likely to lead to the change in outcomes. The realist approach was ideal in unpacking the ‘black box’ of the mechanism, focusing our attention on the role of the context in shaping how the participants engaged with the lay health workers, and whether the intervention achieved its potential. Recent debates have questioned whether a realist evaluation can be combined with a randomised trial.^21–23^ We have found the combination valuable, despite not purposively selecting clinics on the basis of our hypothesis as to what might influence the outcomes (as recommended by the realist approach).

Additional strengths of the study included collecting data prior to, and throughout, the intervention, and working with local fieldworkers experienced in collecting data in a well-established research site. Having a researcher responsible for the process evaluation, and an implementation manager responsible for the intervention, allowed us to separate implementation and research activities, preventing a feedback loop of research information that might have led to changes in the intervention.

We experienced the Hawthorne effect of having nurses changing from their usual approaches to their work because of having someone in the clinic observing them. We addressed this limitation by having longer observation periods, and nurses reverted to their normal work practices after some time and got used to the observers. Second, we did not purposively select the clinics, as is expected in realist studies, because the research was embedded in a randomised controlled trial. This explains the homogenous nature of the results.

## Conclusion

In the context of growing demand for chronic care, our findings show task shifting of certain tasks from nurses to lay health workers can lead to improvements in the quality of care. Though the integrated chronic disease management initiative has provided a coordinated and integrated approach to chronic care in South African primary care clinics, it has created an additional workload for nurses. Introducing lay health workers improved the functioning of this government initiative. However, if a task shifting intervention is to achieve its full potential, local managers need to be able to respond to the constantly changing system, deal with space and equipment challenges, prevent potential conflict between staff and not overwhelm the lay health workers with additional tasks. In a resource-constrained environment, it is important to strengthen facility-level management and leadership skills. Clinic-based lay health workers are an important new resource to manage the rapidly increasing demand.
