# Supplementary material for: Task shifting to improve the provision of integrated chronic care: realist evaluation of a lay health worker intervention in rural South Africa
Source: BMJ Glob Health. 2019 Jan 29;4(1):e001084. doi: 10.1136/bmjgh-2018-001084 (PMC6352781; doi:10.1136/bmjgh-2018-001084)
Supplement: Supplementary data [file bmjgh-2018-001084supp001.pdf]

Data collection tools

1. Clinic observation framework

1. Name of facility	
2. Name of person making observations	
3. Date	

Notes to observers

- Detailed field notes must be kept for each visit. This tool includes a list of issues for you to consider in the observation. Please write detailed descriptions of what you see. In places you will also need to answers the questions below.
- During the observation, detailed attention should be paid to the 'who, what, where and when' of the different processes taking place at the facility. Here, a detailed description of the steps an average patient goes through from arrival to leaving the health facility. The what, with whom, where and when should be derived from observing several individual patients and should be summarized, e.g. in tabular form.
- After these more general observations, the focus of the observation should shift to the interactions between people in the facility (providers and patients, providers and each other, patients and each other).
- This is not JUST a questionnaire. Rather, it is a set of categories/themes to guide the observation process.

**Pathway of care**	**Problem along pathway that leads to loss of patient from care**
**Step 1**: Hypertensive patient in the community	**Problem 1:** Doesn't go to clinic for hypertension or for any other reason
**Step 2:** Patient goes to clinic, for hypertension or another reason	**Problem 2:** BP not measured
**Step 3:** BP taken by receptionist or health care worker	**Problem 3:** BP not recorded;<br>**Problem 4:** Patient and/or nurse not told BP level<br>**Problem 5:** Patient not given medication, adherence counselling, and/or lifestyle advice,<br>**Problem 6:** no return appointment made
**Step 4:** Given diagnosis, medication, and/ or asked to come back for another test	**Problem 4:** Doesn't come back (no money, doesn't think it is serious) **OR** only comes irregularly (because of money, access, ill health, migrant worker)
**Step 6:** Comes back regularly<br>>> but various problems prevent access to care or deter patient from regular attendance	**Problem 6:** Drug supply is erratic;<br>**Problem 7**: Patient file is lost, so don't know patient history;<br>**Problem 8:** Long queues / no drugs<br>**Problem 9:** Nurses are overwhelmed by HIV patients, pay little attention to HT patients;<br>**Problem 10:** Nurses are rude to patients /indifferent to needs to patients
**Step 7:** Comes back regularly	**Problem 11:** Collects but doesn't take pills (BP is not reduced)
**Step 8:** BP is reduced	

MAP OF CHRONIC PATIENT PATHWAY AND STATIONS ALONG THE WAY

Please develop your own KEY. These are some examples:
N=nurse; LHW=lay health worker; DC=Data capturer
eg. LHW (vital signs) = a roving LHW who is doing the vital signs temporarily
eg. LHW (booking) = a LHW who was doing the booking most of the day

PLEASE DESCRIBE WHAT IS HAPPENING ALONG THE PATIENT PATHWAY IN YOUR OWN WORDS

Include patients, clinic staff, how the various forms and files are being used.

PLEASE DESCRIBE THE FOLLOW UP OF PATIENTS WHO DON'T COME ON THEIR APPOINTED DAY

1. Is any follow up done; who does it; how?
2. Please describe the follow up process;
3. What happens when a chronic patient comes when they don't have an appointment

PLEASE DESCRIBE HOW MINOR AILMENT PATIENTS WITH RAISED BLOOD PRESSURE ARE BEING FOLLOWED UP

DESCRIPTION OF THE CHRONIC DISEASE FILING

1. Please describe how the filing system works?
    - Does it work with numbers, or name, or ID
    - Are patients' files pulled out prior to the patient's arrival?
    - Are files filled back again the same day?
    - Describe what the patients carry with them;

2. Are there problems with the filing system that you can see? If yes please describe...
    - What happens if a person doesn't have their card or book?

THE NATURE OF THE INTERACTION: Amongst health workers, and with patients in general

Please describe interactions between staff to illustrate your conclusion

Include an account of the discussions at the staff meeting

DAILY TASKS

Please describe examples

1. How do people go about their jobs on a day-to-day basis (with diligence, calmly, carelessly, distractedly)?
2. Do staff members seem happy, willing, resentful, disinterested, afraid?
3. How often do staff members take breaks? How long are these breaks? Do they take them at the same/different times?
4. How busy do providers seem to be? Are they all equally busy? Are they busy at certain times, or the whole day?
5. Are staff members given 'freedom' to conduct their duties in an uninterrupted way or do supervisors interrupt arbitrarily?

GENERAL CLINIC OPERATION

How many patients were in the queue as the clinic was opening

How many patients are in the queue one hour after opening?

How many patients are in the queue at 11am?

How many patients did the nurse tell to come back tomorrow?

Number of nurses present today?

Is there a chronic care clinic today?

Number of nurses in chronic disease treatment room (s)?

Are there any CHWs at the clinic? How many?

Please describe their activities?

How many working BP machines are there? What type of machines are they (electronic or sphygmomanometer?) If electronic, are replacing the batteries a problem, please describe

Are there both big and small cuffs

MEDICATION

Please describe the system for giving the patients their medication

Remember the difference between objective and subjective notes

1. Is the medication given to patients in the consultation room? ; Is it prepared ready before the patient comes?
2. Who is doing this and where?

Does the clinic have the following drugs

**Name of drug**	**Is this drug in the clinic?**
Hydroclorotiazide (HCTZ, RIDAC)	
Perindopril (Prexum, Coversyl)	
Indapamide (Prexum plus, Coversyl plus)	
Enalapril (Pharmapress)	
Atenolol (Tenblocka)	
Nifedipine (Slow release, Adalat XL, Amloc)	

OTHER OBSERVATIONS

About the context

About the time taken to do some processes

About the processes and interactions

About the actors involved

Other issues?

Patient observation framework

OBSERVATION OF CONSULTATION WITH HYPERTENSIVE PATIENTS

Please complete this check list for 5 consultations with hypertensive patients. Approach a patient in the queue (or ask nurse to introduce you) and ask if you may accompany them. **SOME QUESTIONS YOU WILL HAVE TO ASK THE PAPTIENT DIRECTLY (E.G. ABOUT BP IS MEASURED BEFORE CONSULTATION)**

	**Patient 1**
0. Nurse descriptor (red shoes / braids etc)	
1. Brief description of patient (gender, age,)	
2. whether and who measures blood pressure;	
3. where is this information recorded;	
4. Whether patient was told reading,	
5. whether the reading is explained to the patient,	
6. relevant life style advice is given (reduce salt, exercise, lose weight)	
7. Is medication given?	
8. whether a return appointment is booked;	

**Patient 1**
Describe engagement between nurse and patient, patient’s language, body language, eye contact, facial expression, was nurse concerned about patient?

2. Patient observation framework (consultation length)

OBSERVATION OF NUMBER AND LENGTH OF CONSULTATION WITH CHRONIC PATIENTS

Please complete this form for **ALL** consultations with chronic patients on this particular day. **Sit outside the chronic room. Observe the number of chronic observations made in each chronic room and the length of each consultation.** At the end of the day, confirm with the LHW/chronic care nurse on the total number of booked and unbooked patients for the day. These observations must take place for all the 3 days of clinic observations.

4. Name of facility	
5. Name of person making observations	
6. Day and Date	

Patient No.	Chronic room 1 (time in minutes)		Patient No.	Chronic room 2 (time in minutes)
1.			1.	
2.			2.	
3.			3.	
4.			4.	
5.			5.	
6.			6.	
7.			7.	
8.			8.	
9.			9.	
10.			10.	
11.			11.	
12.			12.	
13.			13.	
14.			14.	
15.			15.	
16.			16.	
17.			17.	
18.			18.	
19.			19.	
20.			20.	
21.			21.	
22.			22.	
23.			23.	
24.			24.	
25.			25.	
26.			26.	
27.			27.	
28.			28.	
29.			29.	
30.			30.	

3. Questionnaire for patient exit structured interviews

AGINCOURT Health & Population Unit, School of Public Health · University of the Witwatersrand, MRC/Wits Rural Public Health and Health Transitions Research Unit	**NKATEKO TRIAL PATIENT EXIT INTERVIEWS**	Questionnaire: Fieldworker: 1 1 1 1 Date: Y Y Y Y M M D D

**0a**	Mbuyelo wa mbhurisano<br>*Interview outcome*	1 = Interview complete<br>**2** = Interview partly completed (write down why not completed)<br>3= Refusal	**0a**	1
**0b**	Comment			

Yana emahlweni na ntirho tani hi laha papilla ra mpfumelelo ri vuleke ha kona.
Now carry out the process of getting informed consent as instructed.

Introduction to the questionnaire: The following questions ask about your experience of care at the clinic.
DO NOT INTERVIEW SOMEBODY WHO HAS COME ON BEHALF OF THE PATIENT

PATIENT EXIT INTERVIEW

1	*Date and time of interview*	yyyy/mm/dd/ hr/mins	1	Y Y Y Y M M D D<br>h h m m
2	*Vito ra kliniki*<br>*Clinic name (interviewer to complete)*	1= Agincourt<br>2 = Arthurstone<br>3 = Belfast<br>**4** = Cork<br>**5** = Cunningmore<br>**6** = Kildare<br>**7** = Lillydale<br>**8** = Justicia<br>**9** = Xanthia	2	1
3	*Rimbewu*<br>*Gender (interviewer to complete)*	**1 =** male ; **2 =** female	3	1 1
4	*Xana u na malembe mangani?*<br>*How old are you?*	Age in completed years **(write 00 if not known)**	4	1 1
5	*Xana u nga va u tiva nkarhi lowu u teke hi wona laha kliniki namunthla?*<br>*Do you know what time you came to the clinic today?*<br>*(probe if respondent unsure using questions like "Were you here when the clinic opened?" or "Were you here when there was health talk in the morning?")*	hrs/mins<br>**(write 99 99 if respondent doesn't know)**	5	h h m m
5a	*Xana mungheneleri a ti tshemba ku fika kwihi mayelana na nkarhi lowu a fikeke hi wona ekliniki?*<br>*How confident the respondent was in relation to time of arrival to the clinic?*	1=respondent confident of the time<br>2=respondent uncertain<br>3=respondent unable to give time at all	5a	1
6	*Xana mabelo ya wena ya mbilu ma pimiwile namunthla?*<br>*Was your blood pressure measured today?*	1 = Yes **2** = No	6	1
7	*Xana u kumile marungula hi riqingho ku tsundzuxiwa leswaku u ta ekliniki namunthla ku suka eka un'wana wa vatirhi va laha kliniki?*<br>*Did you receive an SMS reminding you to come to the clinic today?*	1 = Yes **2** = No	7	1
8	*Xana u nga va u foneriwile ku tsundzuxiwa leswaku u ta ekliniki namunthla ku suka eka un'wana wa vatirhi va laha kliniki?*<br><br>*Did you receive a phone call reminding you to come to the clinic today?*	1 = Yes **2** = No	8	1
9	*Xana u n'wana wa vatirhi a nga va a vulavurile na wena ku ku tsundzuxa leswaku u endzela kliniki namuntlha?*<br>*Did someone from the clinic speak to you reminding you to come to the clinic today?*	1 = Yes **2** = No **(--->Q11)**	9	1
10	*Xana marungulo hi riqingho,kumbe ku foneriwa na switsundzuxo swi ku pfunile e ku ku tsundzuxeni ku ta ekliniki?*<br>*Was the SMS/phone call/reminder helpful in reminding you to come to the clinic'*	1 = Yes **2** = No	10	1
11	*Xana u na nkarhi wo fika kwihi u ri karhi u teka vutshunguri bya mavabyi ya high blood?*<br>*How long have you been receiving treatment for hypertension?*	**1** = Less than 6 months<br>**2** = From 6 months to less than 1 year<br>**3** = 1 year to less than 2 years<br>**4** = 2 years to less than 5 years<br>**5** = 5 years and above	11	1
Xana u vulavurile na mutirhi wa swa rihanyu mayelana na swin'wana swa leswi swi landzelaka?(hlaya nxaxameto)<br>*Did you talk to a health worker today about any of the following? (read out list)*				
12	*Swilo leswi u swi dyaka?*<br>*The things you eat*	1 = Yes **2** = No	12	1

**13**	*Ku hunguta mpimo wa munyu eka swakudya swa wena?*<br>*Reducing the amount of salt in your food?*	**1** = Yes **2** = No	**13**	1
**14**	*Ku endla vutiolori?*<br>*Doing exercise*	**1** = Yes **2** = No	**14**	1
**15**	*Maphilisi/vutsunguri lebyi u byi tekaka na leswi u faneleke ku tekisa xiswona?*<br>*The pills you take and how you should take them*	**1** = Yes **2** = No	**15**	1
If ALL questions from Q12 to Q15 the response is NO, skip to Q17				
**16**	*Xana u ehleketa leswaku u ta cinca swin'wana swa leswi u swi endlaka hikwalaho ka leswi mutirhi wa swa rihanyu a ku nyikeke swona?*<br>*Do you think you will change anything you do because of the advice the health worker gave you?*	**1** = Yes **2** = No	**16**	1
**17**	*Xana u vone onge mutirhi wa swa rihanyu a ri na ku khumbeka leswaku u van a rihanyu lerinene?*<br>*Did you feel the health workers cared that you have good health?*	**1** = Yes **2** = No	**17**	1
**18**	*Xana ku nga va ku ri na vutshunguri/mirhi yo karhi leyi muongori a fanele a ku nikile kambe yi nga ri ki kona laha kliniki?*<br>*Is there any medication that the nurse should have given you but is out of stock?*	**1** = Yes **2** = No	**18**	1
**19**	*Xana masiku ya wena ya ku endzela laha kliniki nakambe ma rini?*<br>*When is your return date?*	Y Y Y Y M M D D		
QUESTIONS FOR PATIENTS AT INTERVENTION CLINICS only<br>Sweswi ndzi lava ku ku vutisa swivutiso swin'wana mayelana na vapfuneti va swa rihanyu. I tintombhi leti mbaleke (muhlovo) wa mahembe.<br>Now I want to ask you some questions about the lay health workers in the clinic. They are the ladies wearing the (colour) shirts				
**20**	*Xana u twile tidyondzo ta swa rihanyu ku suka eka mupfuneti wa swa rihanyu namunthla?*<br>*Did you hear a health talk from a lay health worker today?*	**1** = Yes **2** = No	**20**	1
**21**	*Xana u vulavurile na mupfuneti wa swa rihanyu namunthla?*<br>*Did you talk to a lay health worker today?*	**1** = Yes<br>**2** = No **(end of questionnaire)**	**21**	1
Xana I yini leswi u swi vulavuleke na mupfuneti wa swa rihanyu? (hlaya nxaxameto)<br>*What did you talk to the **lay health worker about?(READ OUT THE LIST)***				
**22**	*Ku va mabelo ya mbilu ya wena ma pimiwa?*<br>*Having your blood pressure measured?*	**1** = Yes **2** = No	**22**	1
**23**	*Ku endla siku ro vuyela ekliniki nakambe?*<br>*Making an appointment to come again?*	**1** = Yes **2** = No	**23**	1
**24**	*Ku landza vutshunguri bya wena?*<br>*Collecting your medication?*	**1** = Yes **2** = No	**24**	1
**25**	*Swakudya leswi u swi dyaka?*<br>*The things that you eat?*	**1** = Yes **2** = No	**25**	1
**26**	*Ku hunguta mpimo wa munyu eka swakudya swa wena?*<br>*Reducing the amount of salt in your food?*	**1** = Yes **2** = No	**26**	1
**27**	*Ku endla vutiolori?*<br>*Doing exercise?*	**1** = Yes **2** = No	**27**	1
**28**	*Maphilisi/vutsunguri lebyi u byi tekaka na leswi u faneleke ku tekisa xiswona?*<br>*The pills you take and how you should take them?*	**1** = Yes **2** = No	**28**	1
**29**	*Xana ku nga va ku ri na swin'wana?*<br>*Anything else the Lay health worker talked about?*	**1** = Yes **(give details)**<br>**2** = No **(---> Q31)**	**29**	1
**30**	Comment			
**31**	*Xana u ehleketa leswaku u ta cinca swin'wana swa leswi u swi endlaka hikwalaho ka leswi mutirhi wa swa rihanyu a ku nyikeke swona?*<br>*Do you think you will change anything you do because of the advice the lay health worker gave you?*	**1** = Yes **2** = No	**31**	1
**32**	*Xana u nga va u twe onge mupfuneti wa swa rihanyu a ri na ku khumbeka leswaku u va na rihanyu lerinene? (nhlamulo yin'we)*<br>*Did you feel the lay health worker cared that you have good health? (single answer)*	**1** = Yes **2** = No	**32**	1
**33**	*Is the patient **booked** or **unbooked**?*	**1** = booked<br>**2** = unbooked	**33**	1

4. Guide for monthly in-depth interviews with lay health workers

Main question: Please can you tell me what has been happening in the clinic?
Aim with this main question is to get the person talking...so may need to be silent and wait for them to talk. Once the LHW has said what is on their mind...then you can ask some more specific questions, such as:

- Can you tell me what have been your main activities this last month?
- Have they changed from the previous months? Why did you make these changes?
- Let's discuss each of those activities in detail.... (also ask to see forms/ card/filing system how and how it is working)
- What about non-LHW activities you have been engaged with in the clinics and why were you involved in those activities?
- Can you tell me about particular successes you have had in the last week or last month?
- Can you tell me about particular problems or challenges you have had?
- Tell me about how the clinic has been functioning this last week / month.
    - Major / notable events;
    - Shortage of nurses/patient load / appointments;
    - drugs/ equipment/ non-Nkateko CHW;
- Can you tell me about atmosphere/ relationship between different staff,
    - between staff and patients;
    - Staff meetings – how are they conducted and issues discussed

5. Guide for monthly in-depth interviews with the Implementation Manager

- What has been happening this month in X clinic?
- What are you spending your time doing?
- How are the clinics functioning?
- What are the LHWs doing, and is it helping? Successes / challenges?
- What other activities / changes are taking place in the clinic?
- Please describe the involvement of the facility manager

To prompt with the following if topics don't come in conversation:
Performance of LHW;
LWH Taking initiative;
LHW responding to problems;
Activities are co-evolving
Engagement between LHW and patients
Engagement between LHW and nurses;

REPEAT FOR EACH CLINIC

6. Interview guide for semi-structured interviews with clinic operation managers

<u>Questions for clinic managers in intervention clinics only</u>

I would like to hear from you, your experience with the programme - the roles of the LHWs, their performance, successes, changes, and challenges experienced in the clinic. What are the main contributions the LHWs and the programme in general, has made to the clinic

- Contributions
- Challenges
- Performance of LHW
- Engagement LHW and patients; LHW and nurses
- Role of implementation manager
- Change over time.
- If the programme was to be re-introduced, what would you recommend to change in its design and approach? And how?

<u>Questions for clinic managers in intervention and control clinics</u>

<u>Staffing level:</u> Staff working at the facility (even if not present on day of interview)

Number	Position / qualification	If attended recent training (if so what)	Responsibilities	Notes (if for example actual activities differ)

- What is your comment on the current level of staff in the clinic
- Last year (2014), there were stories of nurses resigning to get early pension. How did that affect your clinic?
- How does a clinic motivate to be allocated a nurse? Who makes the decision?
- What works well/ not so well with performance appraisals?
- PMDS – do nurses respond at individual or clinic level? Are they expected to put up same answers?

<u>Chronic care</u>

I would like to learn from you how chronic care is provided in this clinic. Every clinic has an 'ideal plan' of how things are meant to work, of what province wants to see when they come. But we all know in reality that life in a clinic is difficult. Sometimes you have enough nurses, sometimes you don't… even with the best will in the world, things don't go as you would like. So in answering my questions I would really appreciate it if you tell me your challenges and struggles…not just what you would like to achieve or where you think the district wants the clinic to be…but what really happens. So, how is chronic care provided in the clinic/ how are chronic patients managed? *Please probe if the following does not come out;*

Appointment system – what works well and what doesn't?

- Is there an appointment system in the clinic? Can you show me? Who is responsible?
- Are you able to follow up patients who don't come back regularly? If so how...
- Are chronic patients booked to come on every day of the week...or on specific days of the week?
- Are hypertensive patients booked for Doctor's review? How often? What happens when a patients does not go for doctor's review? Does the clinic continue to give more medication to the patient? And how long for?

Filing systems- what works well and what doesn't?

- How does the filing system work? Who is responsible? Are files pre-retrieved?
- What is the history of the filing systems in the clinics?
- What are Wits DTs generally doing in the clinic? How are they supporting the clinic?
- What happens if there is no any other space in the files for patients?
- How is the supply of files in the clinic? What happens when the clinic runs out of files?
- What happens when a photocopier runs out of ink, or breaks down?

Queuing/ chronic pathway - what works well and what doesn't?

- What does the receptionist do, if there is one?
- How does the vital signs station operate? Who is responsible?
- Can you explain how the chronic disease room operates? ...explain for HIV patients, TB patients, and hypertensive patients?

Patient management

- Explain to me the standard procedure of managing a minor patient who has been found with elevated BP.
- How helpful has PC 101 been? How confident are the nurses in using it?
- When did the hospitals start moving patients to clinics? What has been the impact? What had been the extra demand?

Difficulties from the health system

- What works well/ not so well with drug supply for chronic patients?
- What works well/ not so well with the referral system?
- What works well/ not so well with supply and maintenance of equipment including BP machines?
- What do they do when equipment breaks down? How do they get things repaired? Do they have any routine equipment maintenance

Role of community health workers (CHW)

- Are there any CHW associated with the clinic? How many? What activities do they do?
- Do some CHW conduct medical in the clinics like dressing wounds, taking vital signs (blood pressure, weight), If yes, have the CHW received related training?
- Do some CHW conduct administrative related activities e.g. filing?
- Who are they answerable to? Who pays them? DoH or NGO?
- What problems do you face with respect to the CHW programme?
- Apart from HIV counselling and testing, what other activities are performed by lay counsellors in this clinic?

Conclusion

- What are other difficulties you face in providing chronic are?
- Is there anything else to tell me?

7. Interview guide for semi-structured interviews with clinic supervisors, sub-district staff & PHC programme staff

Instructions to Interviewer

In this interview please can you describe the overall subject of the interview to the respondent, using the "grand tour" question? Once you have broadly informed the respondent what you are interested in, the respondent is then free to choose which sections they wish to talk about, which issues are most relevant to them. With this more open structure, it is easier to for respondents to describe specific events or examples.

Grand tour question
I would like to understand your experience and views of the Lay Health Worker programme. For example, tell me about how the clinics are functioning, the differences between clinics with LHWs and those without. Which clinics are doing well and why? What are the challenges and successes in the clinics? For Clinics with LHWs I am particularly interested in hearing what the LHWs are doing. So, what can you say about the LHW programme?

Please probe if the following does not come up;

- Which clinics are doing well and why?
- What the LHWs are doing?
- What the implementation manager is doing?
- What are the challenges / successes?
- Whether the management of the clinic is changing?
- Whether management of hypertension is improving?

Other areas to explore:

- What is the history of the filing systems in the clinics?
- Staffing levels – how does a clinic motivate to be allocated a nurse? Who makes the decision?
- What do they do when equipment breaks down? How do they get things repaired? Do they have any routine equipment maintenance?
- Explain to me the standard procedure of managing a minor patient who has been found with elevated BP.
- What qualifies one to become a clinic manager – any career development programme for the clinic managers?
- I also want to understand your own career history….What path to your current post, and your own experience of being a supervisor/ manager.
